# Supplementary material for: Molecular identification of organic vapors driving atmospheric nanoparticle growth
Source: Nat Commun. 2019 Sep 30;10:4442. doi: 10.1038/s41467-019-12473-2 (PMC6769005; doi:10.1038/s41467-019-12473-2)
Supplement: Supplementary file 1 — Supplementary Information [file 41467_2019_12473_MOESM1_ESM.pdf]

Supplementary Information for

Molecular identification of organic vapors driving atmospheric nanoparticle growth

Mohr et al.

Supplementary Figures

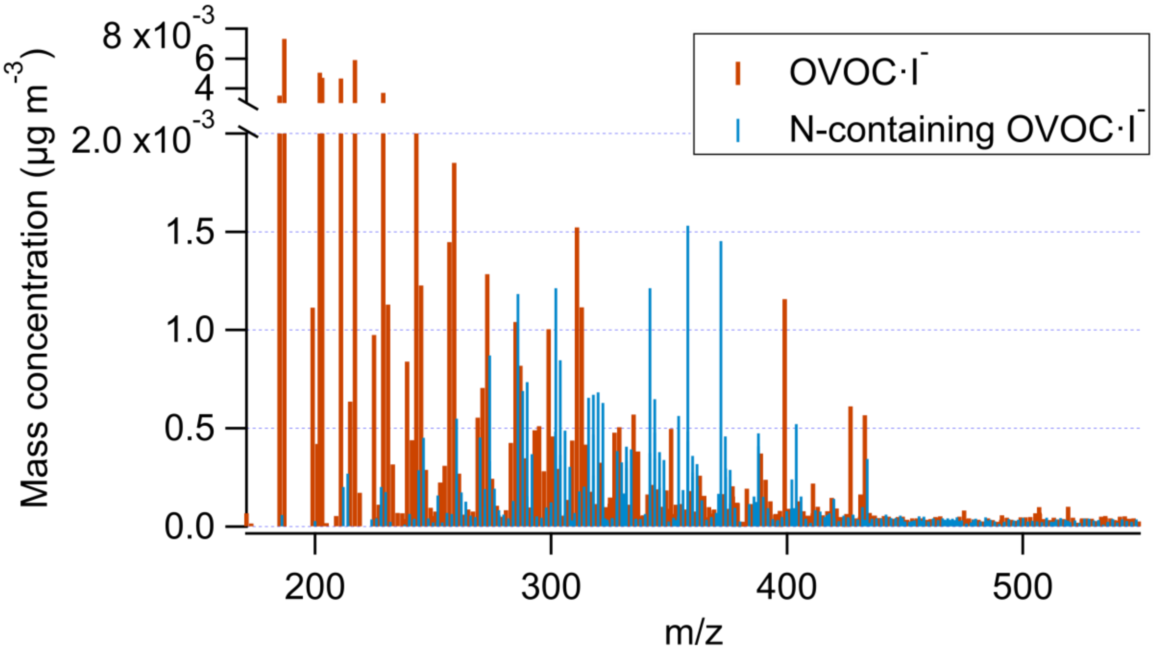

Supplementary Figure 1: Gas phase mass spectrum measured by I-TOF-CIMS. The mass concentrations are the median of all NPF events of this study. OVOC and N-containing OVOC are plotted as their cluster with iodide; the actual compound mass-to-charge ratio (m/z) would thus be shifted by -126.9.

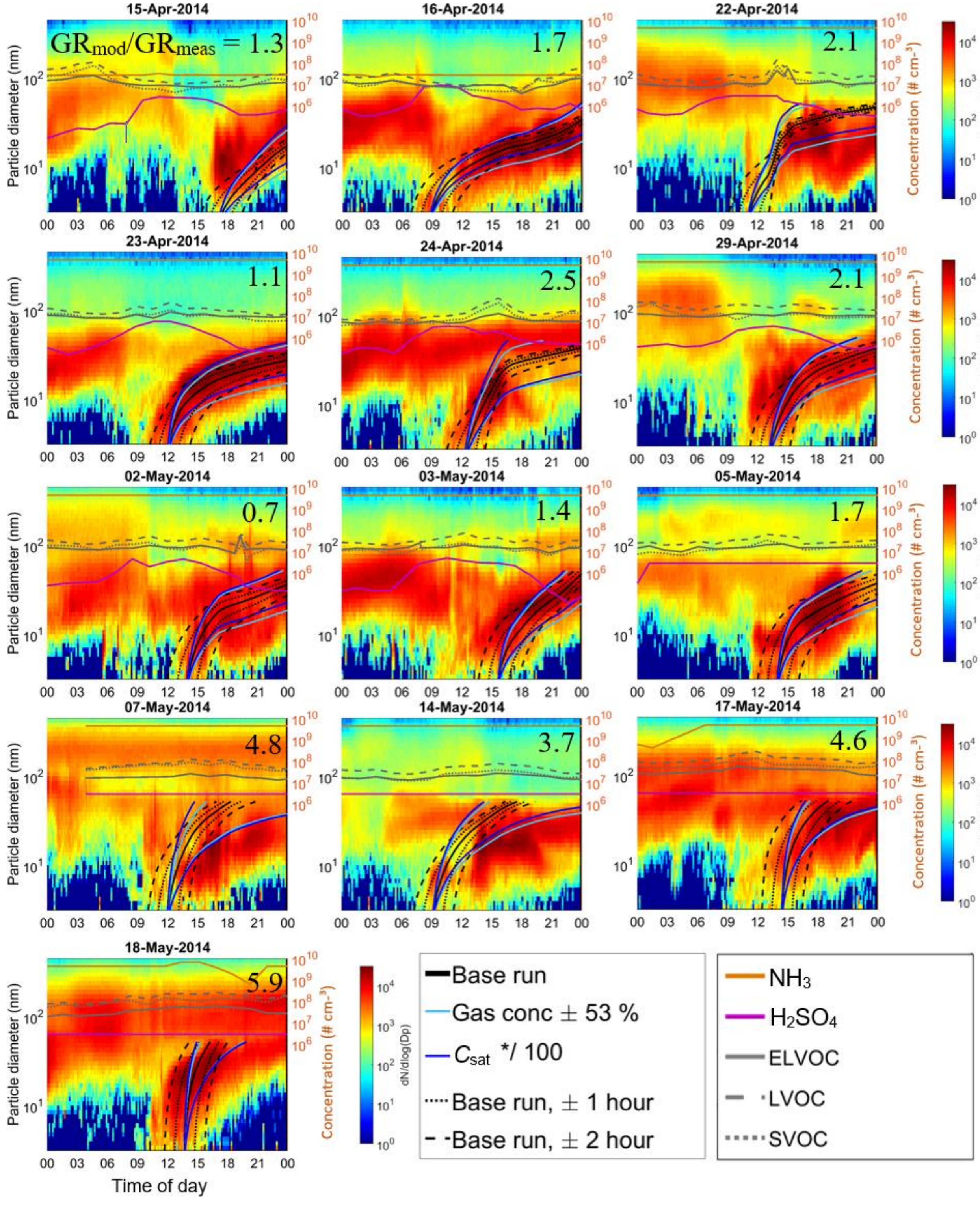

Supplementary Figure 2: Modelled and measured particle growth. Modelled growth using OVOC measured by I-TOF-CIMS as model input on top of measured size distributions by DMPS for all days (24 h plot) with events during the April – May 2014 intensive campaign. The numbers

indicate the ratio of modelled to measured growth rates shown in Figure 3 (averaged over the entire period of the simulation run). Measured time series of SVOC, LVOC, ELVOC, sulfuric acid, and ammonia used in the model simulations are added. For NPF events where concentration measurements of ammonia and sulfuric acid are missing, daytime averages (8:00 – 18:00) over the whole measurement period were used.

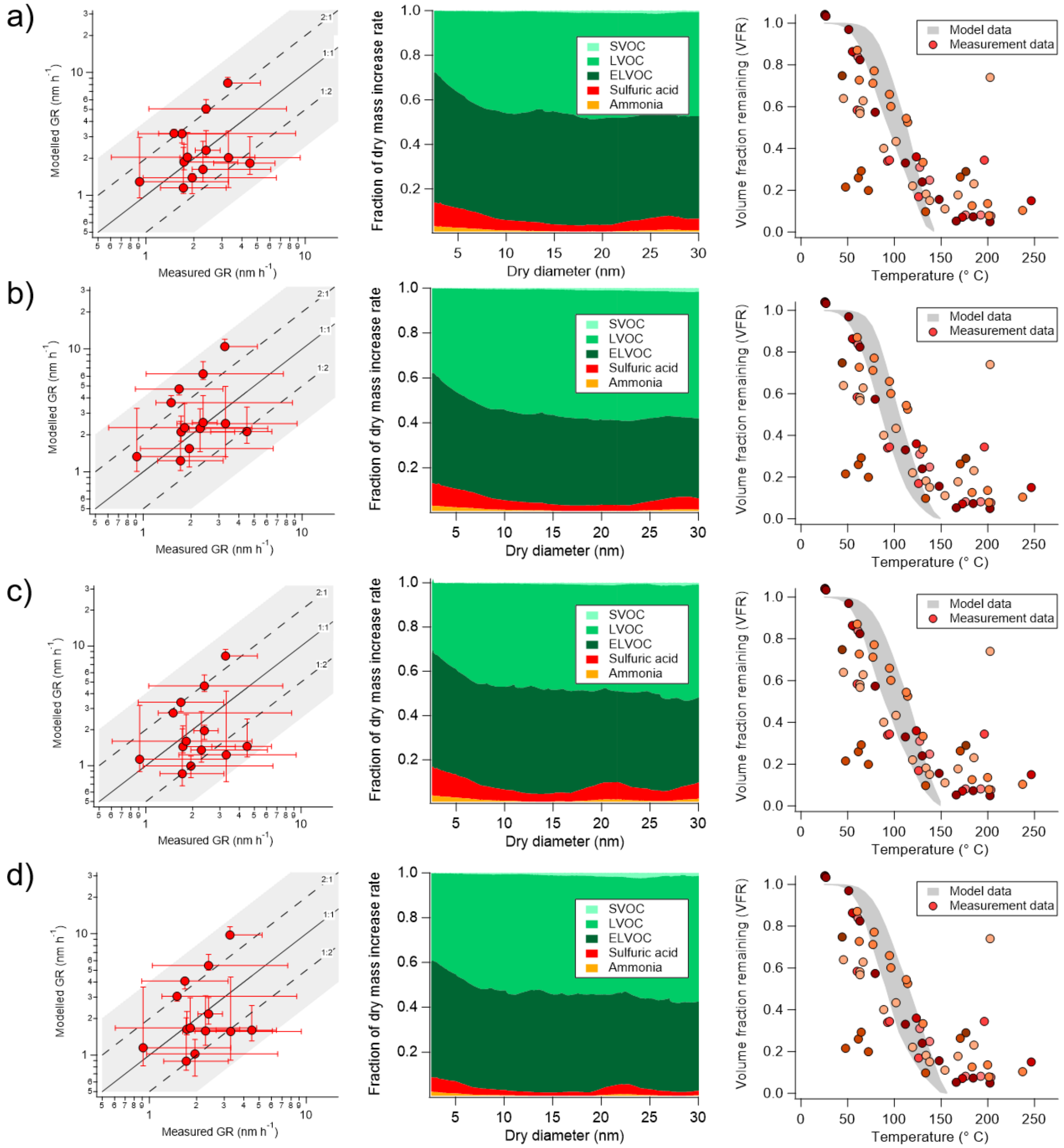

Supplementary Figure 3: Particle phase composition and volatility. Modelled vs measured growth rates (3 – 30 nm) (left panel), average contributions of 15 volatility bins lumped to SVOC, LVOC, and ELVOC to dry particle mass increase as a function of particle size (middle panel), and volume fraction remaining (VFR) of particles with diameter 30 nm measured by TD and modelled using the particle volatility distribution as given by the MABNAG output for all NPF events (right panel), using a) our C_{sat} parametrization values multiplied by a factor of 100, b) C_{sat} as

parametrized by Stolzenburg et al.[20], c) the lower limit of the uncertanty range of the measured gaseous OVOC concentrations (-53%), and d) using a mass accommodation coefficient α_M of 0.5. In the left panels, modelled GR are calculated over the growth from 3 to 30 nm by a linear fit to the diameter as a function of time. The error bars for the modelled GR represent the variation in GR if it is calculated as a fit to different size ranges (3-5 nm, 5-10 nm, 10-20 nm, 20-30 nm). The measured GR are calculated from particle size distributions by a linear fit to the nucleation mode peak diameter as a function of time. The error bars for the measured GR represent the variation in the growth rate when the linear fit is performed on mode peak diameters from different time intervals of the observed nanoparticle growth.

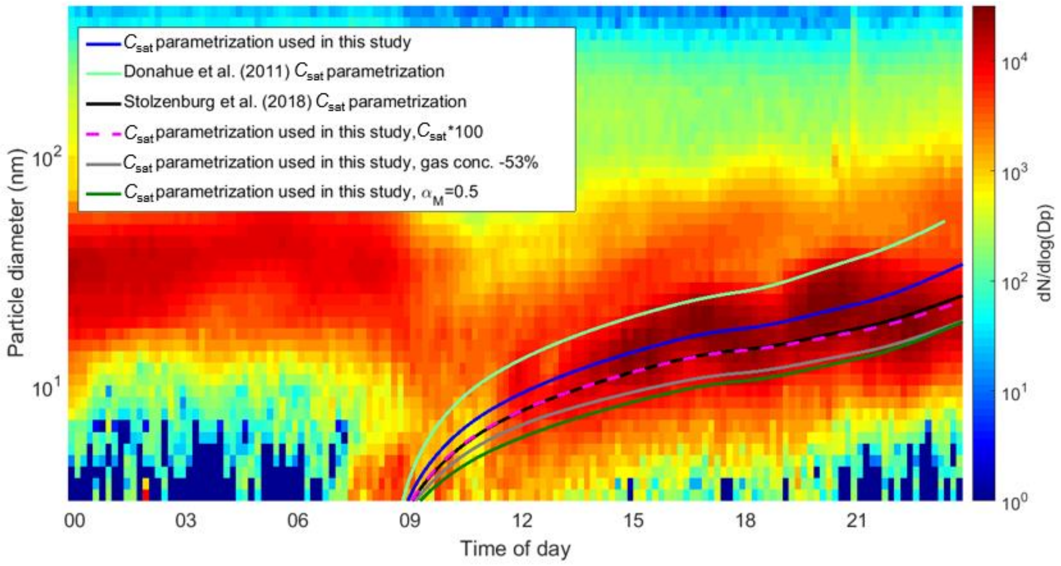

Supplementary Figure 4: Sensitivity study of modelled growth rates. Modelled growth rates for NPF on April 16, 2014, using three different C_{sat} parametrizations, our C_{sat} parametrization with values multiplied by a factor 100, gas phase concentrations lowered by 53%, and α_M = 0.5 on top of size distributions measured by DMPS.

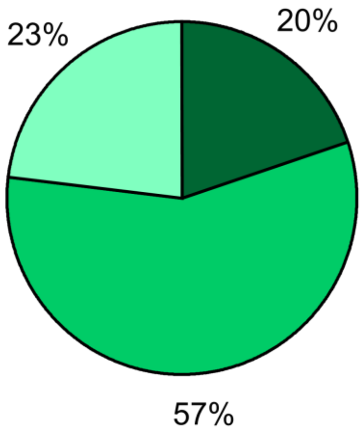

Supplementary Figure 5: Measured volatility distribution in the aprticle phase. Volatility distribution (20% ELVOC, 57% LVOC, 23% SVOC) of mass of OVOC compounds measured in the particles phase by an I-TOF-CIMS with a Filter Inlet for Gases and AEROsols (FIGAERO) and a Microorifice Uniform Deposit Impactor (MOUDI) at the top of the particle phase inlet at the same location and season, but a different year (2013) and acetate reagent ion chemistry (Methods).
